# Multiple-locus variable-number tandem-repeat analysis of S*treptococcus pneumoniae* and comparison with multiple loci sequence typing

**DOI:** 10.1186/1471-2180-12-241

**Published:** 2012-10-22

**Authors:** Hélène van Cuyck, Bruno Pichon, Philippe Leroy, Alexandra Granger-Farbos, Anthony Underwood, Bruno Soullié, Jean-Louis Koeck

**Affiliations:** 1 HIA Robert Picqué, Villenave d’Ornon, France; 2 Health Protection Agency, Microbiology Services – Colindale, London, UK

**Keywords:** *S. pneumoniae*, MLST, MLVA, Universal marker set, Population structure

## Abstract

**Background:**

*Streptococcus pneumoniae* infections remain a major cause of morbidity and mortality worldwide. The diversity of pneumococci was first evidenced by serotyping of their capsular polysaccharides, responsible of virulence, resolving into more than 93 serotypes. Molecular tools have been developed to track the emergence and the spread of resistant, hyper virulent or non-vaccine type clones, particularly DNA-based methods using genetic polymorphism. Pulsed-Field Gel Electrophoresis analysis (PFGE) and Multiple Loci Sequence Typing (MLST) are the most frequently used genotyping techniques for *S. pneumoniae.* MLST is based on sequence comparison of housekeeping genes clustering isolates within sequence types. The availability of genome sequence data from different *S. pneumoniae* strains facilitated the search for other class of genetic markers as polymorphic DNA sequences for a Multiple-Locus Variable-Number Tandem-Repeat Analysis (MLVA). This study aims at confirming the relevance of MLVA of S. pneumoniae, comparing MLST and MLVA performances when discriminating subgroups of strains belonging to the same Sequence Type (ST), and defining a restricted but universal set of MLVA markers that has at least the same discriminatory power as MLST for *S. pneumoniae* by applying marker sets used by different authors on 331 isolates selected in UK.

**Results:**

A minimum spanning tree was built including the serotypes distribution and comparing MLVA and MLST results. 220 MLVA types were determined grouped in 10 Sequence Types (ST). MLVA differentiated ST162 in two clonal complexes. A minimal set was defined: ms 25 and ms37, ms17, ms19, ms33, ms39, and ms40 including two universal markers. The selection was based on MLVA markers with a Diversity Index >0.8 and a selection of others depending of the population tested and the aim of the study. This set of 7 MLVA markers yields strain clusters similar to those obtained by MLST.

**Conclusions:**

MLVA can discriminate relevant subgroups among strains belonging to the same ST. MLVA offers the possibility to deduce the ST from the MLVA Type. It permits to investigate local outbreaks or to track the worldwide spread of clones and the emergence of variants.

## Background

*Streptococcus pneumoniae* infections remain a major cause of morbidity and mortality worldwide, causing diseases which range in severity from otitis media and sinusitis, to pneumonia, septicaemia and meningitis [1]. *S. pneumoniae* is a commensal of the human nasopharynx [2].

The diversity of pneumococci was first evidenced by serotyping of their capsular polysaccharides resolving into more than 93 serotypes [3,4]. However, only 16 serotypes cause approximately 90% of invasive disease worldwide [1,5]. Due to the natural transformability in the pneumococcus, horizontal recombination allows that one serotype can belong to different genotypes, and a single genotype can express different capsule genes, i.e. different serotypes. This phenomenon is known as capsular switching [6,7]. Capsular serotype may be more important than genotype in the ability of pneumococci to cause invasive disease [8], but there are also some other investigations that underline the importance of genotypes as well [9-13].

Molecular tools, particularly DNA-based methods using genetic polymorphism, have been developed to track the emergence and the spread of resistant, hyper virulent clones or shifts in serotype distribution detected for both non-invasive and invasive disease reported before or since the use of heptavalent protein-polysaccharide pneumococcal conjugate vaccine (PCV7), in different countries [14,15]. Among them, Pulsed-Field Gel Electrophoresis analysis (PFGE) [16,17] and Multiple Loci Sequence Typing (MLST) [9] are the most frequently used genotyping methods for *S. pneumoniae.* PFGE is based on restriction enzyme pattern analysis; MLST is a sequence based method targeting 7 housekeeping genes. A *S. pneumoniae* specific MLST scheme targeting *aroE*, *gdh*, *gki*, *recP*, *spi*, *xpt*, and *ddl* was developed [9] together with an online identification page at http://www.mlst.net[18]. PFGE and MLST have been extensively compared [15,17,19] and both have proven their capacity to discriminate efficiently among genotypes. However PFGE lacks, in some extend, of inter-laboratories reproducibility and MLST is expensive thus may be not affordable for large scale studies. Availability of genome data greatly facilitated the search for polymorphic DNA sequences. Among them, polymorphic tandem repeat sequences also called Variable Number of Tandem Repeats (VNTR) are an interesting class of genetic markers; Multiple alleles may be present at a single locus, and size differences are easily resolved by electrophoresis of PCR products. VNTR has proved to be highly relevant for the typing of pathogenic bacterial species (*Haemophilus influenzae*[20]; *Bacillus anthracis*[21]*; Yersinia pestis*[22])*.* A *S. pneumoniae*- Multiple-Locus Variable-Number Tandem-Repeat Analysis (MLVA) scheme was developed with a dedicated web-based database at http:/http://www.mlva.eu[23]. It targets 17 distinct loci and was used initially to characterise pneumococcal isolates from Burkina Faso [24]. Although discriminatory power of MLVA has been demonstrated, the large number of loci included in the scheme may be a limitation for its use on large scale studies (cost, timeframe, large number of samples).

This study aims at confirming the relevance of MLVA of *S. pneumoniae*, comparing MLST and MLVA performances when discriminating subgroups of strains belonging to the same Sequence Type (ST), and defining a restricted but universal set of MLVA markers that has at least the same discriminatory power as MLST by comparing the population genetic structure of *S. pneumoniae* using different published sets of markers [15,19,23,25,26].

## Methods

### Bacterial strains

331 invasive isolates of *Streptococcus pneumoniae* from the Health Protection Agency collection, London, UK, collected during the period 2002–2006, were selected among the 10 major MLST sequence types (STs), circulating in England and Wales (see [27] and [28] for detailed MLST methodology), with approximately 30 isolates per ST. Selection included serotypes commonly associated with these STs and all possible serotype variants (Table 1) identified in the HPA collection. Isolates were serotyped by slide agglutination against the full antisera panel from the Danish Statens Serum Institute (Denmark) as part of the Systemic and Respiratory Infection Laboratory (HPA, London) reference service. The isolates were collected from blood (314), cerebral spin fluid (13), pleural fluid (2), abscess (1), and bronchial aspirate (1).

**Table 1:** **Distribution of the 331*S. pneumoniae*isolates**

**Sequence Types (ST)**	**Serotypes**	**Number of Isolates**		**Singletons**	**MLVA Types (MT)**	**Clonal Complexes (CC)**
		**Per Serotype**	**Total**			
9	8	1	33	0	199	CC8
	19 F	2		0	228, 182	
	14	30		0	199, 304, 375, 280, 337, 272, 367, 350, 365,, 354, 347, 383, 378	
65	18C	1	33	0	209	CC5
	22A	1		0	172,	
	6A	29		0	172, 334, 271, 324, 314, 177, 176, 173, 358, 363, 257	
	6B	2		0	172, 206	
138	6B	30	30	**5**	**234, 330, 276, 251, 268**	CC1
					311, 345, 310, 326, 289, 181, 369, 319, 344, 370, 315, 273, 246, 295, 254, 269, 356, 233, 213, 237, 210, 290, 329	
156	14	4	34	0	299, 249, 190, 229	CC9
	6B	1		0	207	
	9 V	29		**1**	**179**, 184, 204, 192, 343, 168, 188, 187, 169, 277, 198, 113, 328, 352, 171, 200, 278, 279, 183, 189, 205, 174, 175, 287	
162 MC162a	1	1	33	0	364	CC9
	14	1		0	225	
	19 F	14		**3**	**270, 300, 301**, 325, 368, 239, 309, 323, 372, 348, 374, 346, 341	
MC162b	9 V	4		**2**	**263, 281**, 252	CC10
	19 F	1		0	351	
	6B	1		0	227	
	9 V	11		0	265, 306, 245, 258, 293, 336	
176	6B	3	31	0	193, 377	CC11
		27		**3**	**282, 274, 214**, 371, 307, 224, 219, 338, 178, 313, 305, 262, 170, 361, 267, 266, 340, 335, 185, 196, 253, 236, 217, 308	CC4
	6A	1		0	224	CC4
180	14	1	33	0	129	CC7
	19 F	1		0	223	
	3	30		**2**	**331, 296**, 288, 156, 232, 138, 285, 238, 222, 384, 312, 248, 327, 349, 360, 230, 294, 240, 318, 320	
	7 F	1		0	222	
199	15B	7	42	**1**	**180**, 191, 194, 256, 195, 216, 220	CC6
	15C	4		**1**	**243**, 366, 362	
	19A	29		0	235, 283, 231, 203, 297, 316, 302, 317, 376, 292, 379, 333, 359, 242, 259, 244, 355, 284, 260, 339	
	19 F	1		0	197	
	9 N	1		0	203	
227	1	29	32	0	215, 380, 250, 255, 186, 211, 201, 322, 298, 247, 357, 342, 376, 264, 382, 275	CC3
	14	1		0	186	
	6A	2		0	211, 226	
306	1	29	29	0	85, 261, 130, 353, 212, 241, 221, 202, 303, 381	CC2

Bold MTs are referring to singletons.

### Methods

MLVA was performed as previously described [23]. The first 17 VNTRs (Spneu 15 to Spneu 41) were used. The last one (Spneu42) unsuccessfully amplified DNA from the isolates or the reference strains and therefore was avoid in this study. For convenience, the nomenclature “Spneu” meaning *Streptococcus pneumoniae* was replaced by “ms” meaning minisatellite in this paper.

### Data analysis

The genetic diversity was measured by the Hunter-Gaston Diversity Index (DI) on http://www.hpa-bioinformatics.org.uk/cgi-bin/DICI/DICI.pl. A high DI with a narrow confident interval (CI) indicates accurate measurement of a highly variable locus. These loci may be sufficiently variable to be used as an indicator to discriminate between samples or as a starting point for assay development.

The genetic distances between two isolates i and j were calculated as following:

$$\text{d}\left(\text{i,j}\right)\;=\;\frac{\text{No}.\;\text{non-identical markers}}{\text{No}.\;\text{markers used}}$$

One marker difference is equivalent to 15%, 5/7 different is 70%. In our study, the criteria sets provided by either MLVA or MLST analysis consider two strains similar having at least 70% similarity, i.e. a DLV difference. The interest of the method is to quantify the difference.

The minimum spanning trees by MLST using the 7 house keeping genes and by MLVA were constructed using BioNumerics ver. 5.0 with the categorical coefficient. Priority rules were fixed as following: maximum number of i) Single-locus variants (SLVs); ii) SLVs and double-locus variants (DLVs); iii) Maximum neighbour minimum cluster size of two loci (DLV) and 2 ST, when the seven housekeeping gene markers were used by MLST; iv) Maximum neighbour minimum cluster size of two loci (DLV) and 2 MT, when 17 markers were used and one locus (SLV) and 2 MT when 7 markers are used by MLVA.

The Congruence among Distance Matrices MLST/MLVA was calculated in % of difference of the genetic distance between two isolates depending on the number of markers used using Bionumerics ver.5.0 as well.

The Inter-Matrix Difference (IMD) was calculated using the formula below, where d(i,j) is the genetic distance between i and j, and n the number of isolates. Marker numbers refer to Table 2. The lower the IMD value is the closest is the distance matrices given by the two techniques.

$$IMD\;=\;\frac{\sum \left|d{\left(i,j\right)}_{\mathrm{mlva}}-d{\left(i,j\right)}_{\mathrm{mlst}}\right|}{n\;\left(n-1\right)/2}$$

**Table 2:** **Genetic diversity of the 331 isolates of*S. pneumoniae***

**Marker name**	DI*	**95% CI †**	**Marker set by author**			
			**This paper**	**Koeck 2008 [**[19]**]**	**Pichon 2010 [**[26]**]**	**Elberse 2011 [**[25]**]**
			**(A)**		**(B)**	**(C)**
ms15_507bp_45bp_7U	0.607	[0.588-0.626]				+
ms17_167bp_45bp_3U	0.852	[0.847-0.857]	+	+	+	
ms19_663pb_60pb_10U	0.674	[0.658-0.691]	+	+	+	
ms25_426bp_45bp_4U	0.788	[0.779-0.797]	+	+	+	+
ms26_492bp_51bp_6U	0.714	[0.703-0.726]				
ms27_326bp_45bp_3U	0.561	[0.543-0.579]	+			
ms31_594bp_45bp_9U	0.695	[0.683-0.708]				
ms32_280pb_45bp_2U	0.598	[0.585-0.611]				+
ms33_407bp_45bp_2U	0.737	[0.725-0.748]	+	+		+
ms34_239bp_45bp_1U	0.682	[0.670-0.695]			+	
ms35_349bp_49bp_4U	0.572	[0.557-0.587]				
ms36_274pb_45pb_2U	0.793	[0.786-0.801]			+	
ms37_501bp_45bp_7U	0.855	[0.851-0.859]	+	+	+	+
ms38_309bp_45bp_2U	0.557	[0.535-0.578]				+
ms39_275bp_45bp_2U	0.812	[0.804-0.819]	+		+	
ms40_376bp_45bp_3U	0.789	[0.782-0.797]		+		+
ms41_166pb_14pb_2U	0.567	[0.548-0.586]		+		
All markers	0.989	[0.987-0.991]				
Congruence (%)			47.2	59	65.1	43.8

The marker name contains all the numbers necessary to characterize the marker in reference to a given sequenced genome (reference strain, R6). For example, in “ms15_507bp_45bp_7U”, - ms means minisatellite, - 507 bp is the size of the amplification product of this marker; - 45 bp is the size of the repeat unit, - 7 is the number of repeats. Markers used by authors are noticed by a cross (+), authors seven Markers set are noticed as following: (A) this paper, (B) Pichon’s and (C) Elberse’s. The MLST/MLVA congruence in percent by author is indicated at the bottom of the table.

* DI: diversity index. *† CI*: confidence interval.

## Results and discussion

The discriminatory power of MLVA was compared to that of MLST by analysing 331 isolates of *S. pneumoniae* which had been previously serotyped and composed 10 sequence types. The discriminatory power was analysed in two steps: first by the analysis of the population including its composition and the genetic diversity using 17 markers, then by analysing the genetic diversity of this population using sets of 7 markers described by different authors [19,25,26].

**Figure 1:**
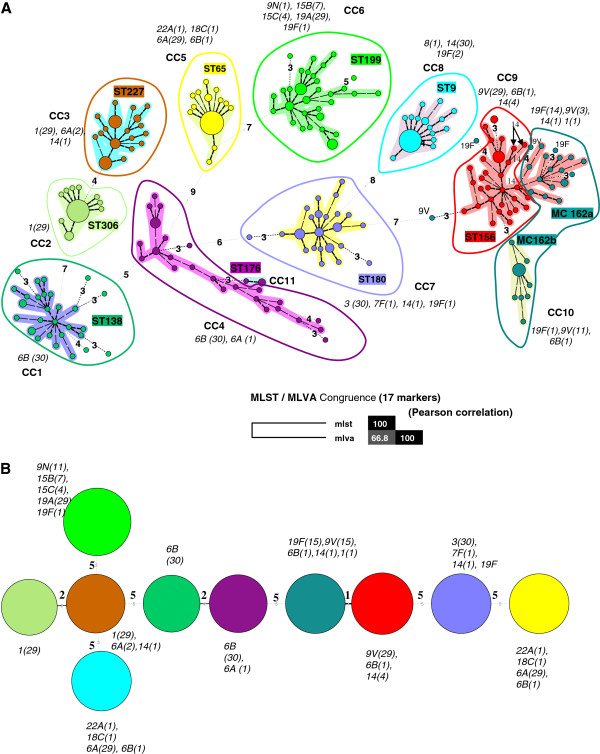
**Comparison of Minimum spanning tree constructed either from 7 MLST markers (housekeeping genes) or from 17 MLVA markers, for 331 *S. pneumoniae *isolates. A:** The minimum spanning tree was constructed with a categorical coefficient. Each coloured circle represents a different MLVA type (MT). The width of the line reflects the genetic distance between ST (heavy short lines connect SLVs, thin longer lines connect DLVs, and dotted lines indicate the most likely connection between 2 STs differing by more than 2 loci). **B:** The minimum spanning tree was constructed with a categorical coefficient. Each circle represents a different MLST type (ST). The colour of a circle and the line clustering the MT with the same colour are corresponding to identical sequence type (ST). Same colours design STs in Figure 1A. Size of the circle reflects the number of isolates designed in italic numbers within parenthesis, while the width of the line reflects the genetic distance between MT (heavy short lines connect SLVs, thin longer lines connect DLVs, and dotted lines indicate the most likely connection between 2 STs differing by more than 2 loci). The number of loci that differ between two MTs is indicated on the lines connecting the MTs. Clonal complexes (CC) were defined as MTs having a maximum distance of changes at 2 loci and a minimum cluster size of 2 types. Each CC as a cluster is shaded in a different colour.

The genetic diversity of the 331 isolates of *S. pneumoniae* was assessed by MLVA by using 17 markers (Table 2). A total of 220 MLVA types (MTs) were identified and clustered into 11 clonal complexes and 17 singletons by minimum spanning tree analysis (Figure 1A). DI > 0.8 was achieved for three loci: ms17, ms37 and ms39, which represent the most discriminatory effect. The congruence between MLST and MLVA was estimated at 67% (Figure 1A). The locus variation using MLST is a DLV between ST227 and ST306, ST138 and ST176, and a SLV between ST156 and ST162 (Figure 1B). Other ST had 5 loci difference. MLVA underlines genetic variability within MLST types. ST9, ST65 and ST 306 are more clonal than the others, whereas ST 176 is much more diversified by MLVA than by MLST, and ST156 and ST162 presented a unique pattern. ST162 is either grouped with ST156 to form a clonal complex or is forming a clonal complex by itself with a 3 locus difference. Isolates of ST162 formed two distinct MLVA complexes (MC), one mainly associated with serotype 19 F (MC162a) and the other one (MC162b) associated with 9 V, suggesting independent evolutionary biology following divergence from a ST162 common ancestor combined with capsular switching event. Moreover, serotype 14, which is an invasive serotype was shown to be a variant of ST156 and 9 V [29], and therefore, was clustered within ST156/162. Other isolates of serotype 14 ST9 are well separated from ST156/162.

Knowing the MLVA type it is possible to deduce not only the ST but also the associated serotype depending on the clonality of the serotypes. It is the case for serotype 1 because of its strong clonality, whereas it is not possible for the serotype 19F. Moreover, the carriage is more frequent for certain serotypes, particularly serotype 19F, meaning that isolates belonging to those serotypes often exchange DNA with other carried. So the serotype of a pneumococcus strain can change but not its other genetic characteristics’. Indeed, carriage serotypes are distributed along the dendrogram and can belong to very different genotypes.

However, in order to compare identical number of MLST and MLVA markers, a set of seven MLVA markers was considered. The set includes three markers with the highest discriminatory power (DI > 0.8), one marker with a low discriminatory power acting as an anchor for the dendrogram, and three others, selected for a low IMD and for their ability to distinguish ST 227 and ST 306, and based on previous data [19]. The composition of the MLVA set was adapted as follows: **ms17, ms19, ms25, ms27, ms33, ms37, ms39***.*

**Figure 2:**
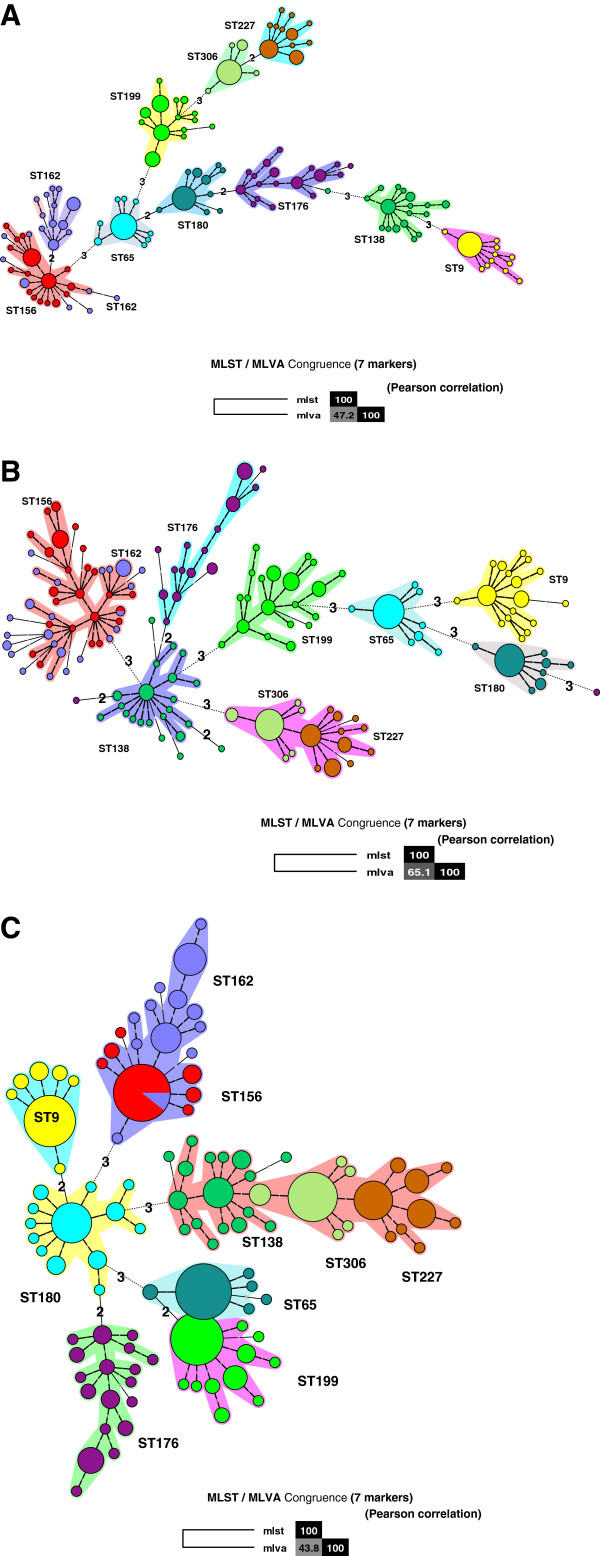
**Minimum spanning tree constructed from 7 MLVA markers for 331 pneumococcal isolates from this study. A:** ms17, ms19, ms25, ms27, ms33, ms37, ms39 markers used for this study; **B**: ms17 ms19, ms25, ms34, ms37, ms39 markers [25]; **C**: ms15, ms25, ms32 ms33, ms37, ms38, ms40 [26]. Clusters were defined as MTs having a maximum distance of changes at 1 loci and a minimum cluster size of 1 type. The minimum spanning tree was constructed with a categorical coefficient. Each circle represents a different MLVA type (MT). The colour of a circle indicates the number of the corresponding sequence type (ST). Size of the circle reflects the number of isolates while the distance between circles reflects the degree of genetic divergence (heavy short lines connect SLVs, thin longer lines connect DLVs, and dotted lines indicate the most likely connection between 2 STs differing by more than 2 loci). The number of loci that differ between two MTs is indicated on the lines connecting the MTs. Each clonal complexes is shaded in a different colour.

The comparison between MLST and MLVA using seven markers was obtained by construction of a minimum spanning tree (Figure 2A). Congruence MLST/MLVA was 47.2%.

Then, congruence between MLST and MLVA of the reduced MLVA scheme was compared to those obtained when using the seven marker set Elberse’s [25] (Figure 2C) and the seven marker set Pichon’s [26] (Figure 2B). Elberse’s scheme was dedicated for studying the population structure of *S. pneumoniae* whilst Pichon’s markers were selected based on the best combination for highest discriminatory power for outbreak investigation. The genetic distance between the 331 isolates determined by MLST and MLVA and their congruence (Figures 2B, 2C and Table 2) was respectively 65.1% (Pichon’s markers), 43.8% (Elberse’s markers). Previously [19], congruence MLST/MLVA was estimated to 59% when the same set of isolates was analysed using markers ms17, ms19, ms25, ms33, ms37, ms40 and ms41. Pichon’s markers gave similar congruence to the 17 marker set of this study, or the highest MLST/MLVA congruence comparing the seven markers sets (A, B, C), but ST227/ST306 and ST156/ST162 were grouped within the same clonal complex. MLST/MLVA results are coherent. Indeed, a low genetic distance between two ST is low between two corresponding MT.

Applying sets of markers selected in two other studies on *S. pneumoniae*, to the population selected in this study, revealed (Table 2) that (i) two markers ms25 and ms37, are commonly used by all authors, including this study, and presented a high DI whichever strains were used and the aim of the study, (ii) several markers were never used: ms26, ms31 and, ms35, (iii) the other markers, ms17, ms19 and ms33 were dependant on the method, i.e., the capacity to discriminate the clonal complexes, (iv) ST discriminant capacity using MLVA varies depending on the set of marker used, and a high percentage of congruence does not mean a better discriminant capacity.

The selection of the markers except for ms25 and ms37 was dependant on the studied population. MLVA based on this study (A), Pichon’s (B), marker sets clustered the study population accordingly to MLST data whilst Elberse’s (C) marker set gave a lower resolving of the population.

The results suggested that 14 out of the 17 markers previously described for *S. pneumoniae,* can be selected whatever the *S. pneumoniae* population considered.

In other words, analysis of strains with the same ST but isolated in different countries will give similar results, i.e., many new MLVA types associated with the same ST can be identified as it was observed for Niger strains [30] (Additional file 1). However, higher the number of markers is, more important the diversity of genotypes observed is. Some markers are specific to the bacterial population [23].

MLVA can discriminate relevant subgroups among strains belonging to the same ST, and offers the possibility to deduce the ST from the MT.

## Conclusion

In this study MLST and MLVA were compared for their discriminatory power for *S. pneumoniae* populations with purpose to try to define a set of marker that can be used whatever the population and the aim of the study.

The study population was composed by 331 isolates belonging to the top 10 STs in England. MLVA using 17 markers yields clustering of the isolates similar to that obtained by MLST. Moreover, MLVA permits to differentiate within ST different clonal complexes, particularly ST156 and ST162. Our study showed that the number of VNTR loci may be reduced to 7 to achieve a similar cluster pattern to MLST.

In conclusion, prior to any study, 14 markers only, have to be tested. Then, the selection of 7 markers is based on MLVA markers with a DI > 0.8 (including markers ms25 and ms37) and a selection of others including one marker with a low discriminatory power acting as an anchor for the dendrogram, and 4 others depending of the population tested and the aim of the study. The set of markers, whose composition depends on the population studied, could be used either to investigate local outbreaks or to track the worldwide spread of clones and particularly the emergence of variants.

## Abbreviations

*S. pneumoniae*: *Streptococcus pneumoniae*; PFGE: Pulsed-Field Gel Electrophoresis analysis; MLST: Multiple Loci Sequence Typing; DNA: Deoxy nucleic acid; MLVA: Sequences for a Multiple-Locus Variable-Number Tandem-Repeat Analysis; MT: MLVA type; ST: Sequence Type; VNTR: Variable Number of Tandem Repeats; ms: Minisatellite; DI: Hunter-Gaston Diversity Index; CI: Confident interval; SLVs: Single-locus variants; DLVs: Double-locus variants; IMD: Inter-Matrix Difference; CC: Clonal Complex.

## Competing interests

MLST testing was funded by a UK Department of Health Grant. MLVA testing was funded by the French Military Health Service. Financial competing interest: Non-financial competing interests. No stocks hold or share in an organization that may in any way gain or lose financially from the publication of this manuscript, either now or in the future. No holding or currently applying for any patents relating to the content of the manuscript. No reimbursements, fees, funding, or salary have been received from an organization that holds or has applied for patents relating to the content of the manuscript. No non-financial competing interests (political, personal, religious, ideological, academic, intellectual, commercial or any other).

## Authors’ contributions

HvC participated to the methodology comparison and drafted the manuscript. BP participated in the design of the study, performed the MLST, provided the isolates and revised the manuscript critically for important intellectual content. PL conducted and carried out the MLVA protocol. AGF carried out MLVA and molecular genetic data analysis and help to draft the manuscript. AU performed the statistical analysis and revised the manuscript. BS revised the manuscript critically for important intellectual content. JLK conceived of the study, and participated in its design and coordination. All authors read and approved the final manuscript.

## Supplementary Material

Additional file 1: Genetic diversity of pneumococcus isolates from meningitis cases in Niger, 2003-2006. (Article in French). Click here for file
