# Supplementary material for: Multiple-locus variable-number tandem-repeat analysis of Streptococcus pneumoniae and comparison with multiple loci sequence typing
Source: BMC Microbiol. 2012 Oct 22;12:241. doi: 10.1186/1471-2180-12-241 (PMC3562504; doi:10.1186/1471-2180-12-241)
Supplement: Additional file 1: — Genetic diversity of pneumococcus isolates from meningitis cases in Niger, 2003-2006. (Article in French). [file 1471-2180-12-241-S1.ppt]

## Slide 1
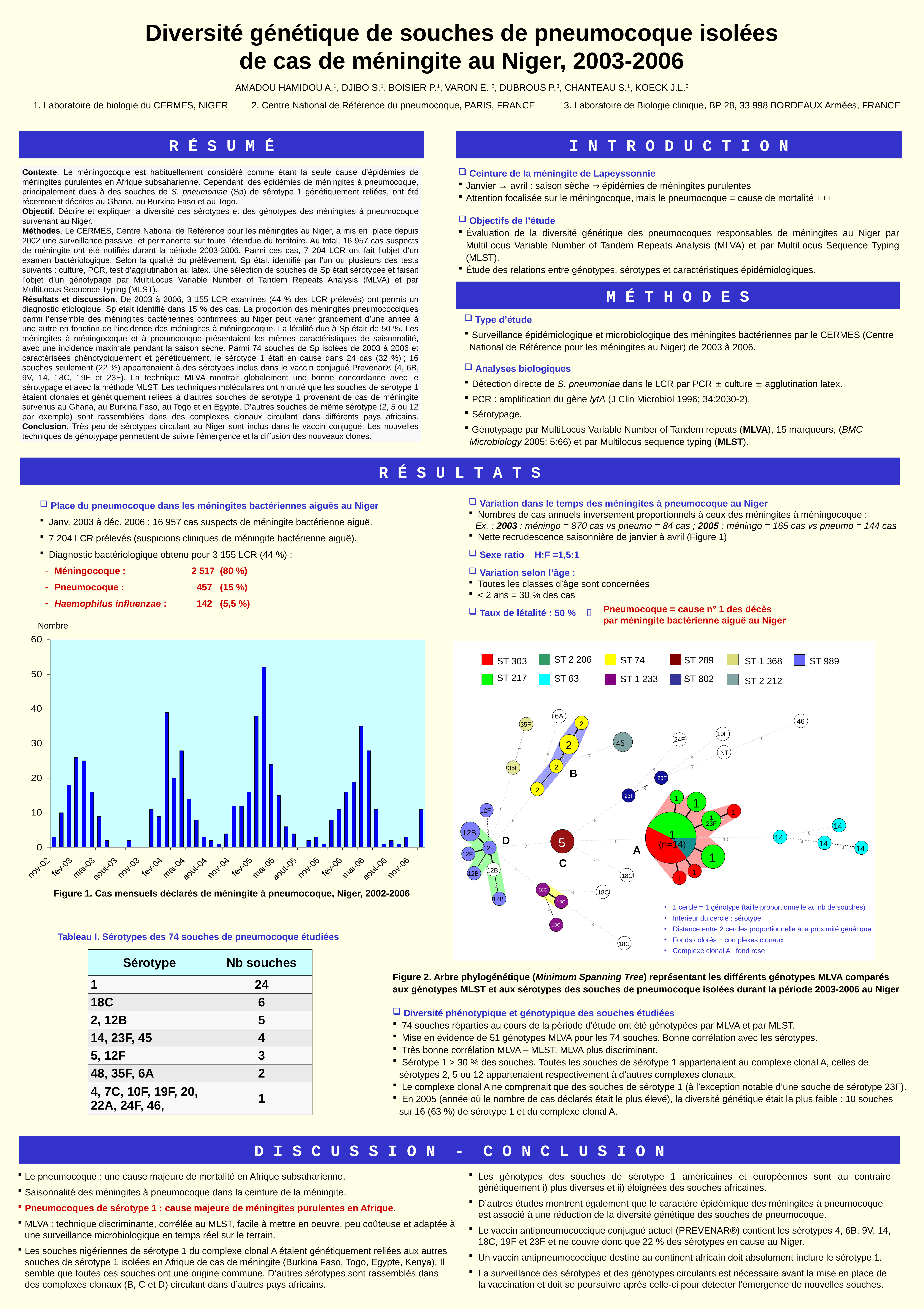

Diversité génétique de souches de pneumocoque isolées
de cas de méningite au Niger, 2003-2006
AMADOU HAMIDOU A.1, DJIBO S.1, BOISIER P.1, VARON E. 2, DUBROUS P.3, CHANTEAU S.1, KOECK J.L.3
1. Laboratoire de biologie du CERMES, NIGER		2. Centre National de Référence du pneumocoque, PARIS, FRANCE		3. Laboratoire de Biologie clinique, BP 28, 33 998 BORDEAUX Armées, FRANCE
R É S U M É
I N T R O D U C T I O N
 Ceinture de la méningite de Lapeyssonnie
Janvier → avril : saison sèche  épidémies de méningites purulentes
Attention focalisée sur le méningocoque, mais le pneumocoque = cause de mortalité +++
 Objectifs de l’étude
Évaluation de la diversité génétique des pneumocoques responsables de méningites au Niger par MultiLocus Variable Number of Tandem Repeats Analysis (MLVA) et par MultiLocus Sequence Typing (MLST).
Étude des relations entre génotypes, sérotypes et caractéristiques épidémiologiques.
Contexte. Le méningocoque est habituellement considéré comme étant la seule cause d’épidémies de méningites purulentes en Afrique subsaharienne. Cependant, des épidémies de méningites à pneumocoque, principalement dues à des souches de S. pneumoniae (Sp) de sérotype 1 génétiquement reliées, ont été récemment décrites au Ghana, au Burkina Faso et au Togo.
Objectif. Décrire et expliquer la diversité des sérotypes et des génotypes des méningites à pneumocoque survenant au Niger.
Méthodes. Le CERMES, Centre National de Référence pour les méningites au Niger, a mis en place depuis 2002 une surveillance passive et permanente sur toute l’étendue du territoire. Au total, 16 957 cas suspects de méningite ont été notifiés durant la période 2003-2006. Parmi ces cas, 7 204 LCR ont fait l’objet d’un examen bactériologique. Selon la qualité du prélèvement, Sp était identifié par l’un ou plusieurs des tests suivants : culture, PCR, test d’agglutination au latex. Une sélection de souches de Sp était sérotypée et faisait l’objet d’un génotypage par MultiLocus Variable Number of Tandem Repeats Analysis (MLVA) et par MultiLocus Sequence Typing (MLST).
Résultats et discussion. De 2003 à 2006, 3 155 LCR examinés (44 % des LCR prélevés) ont permis un diagnostic étiologique. Sp était identifié dans 15 % des cas. La proportion des méningites pneumococciques parmi l’ensemble des méningites bactériennes confirmées au Niger peut varier grandement d’une année à une autre en fonction de l’incidence des méningites à méningocoque. La létalité due à Sp était de 50 %. Les méningites à méningocoque et à pneumocoque présentaient les mêmes caractéristiques de saisonnalité, avec une incidence maximale pendant la saison sèche. Parmi 74 souches de Sp isolées de 2003 à 2006 et caractérisées phénotypiquement et génétiquement, le sérotype 1 était en cause dans 24 cas (32 %) ; 16 souches seulement (22 %) appartenaient à des sérotypes inclus dans le vaccin conjugué Prevenar® (4, 6B, 9V, 14, 18C, 19F et 23F). La technique MLVA montrait globalement une bonne concordance avec le sérotypage et avec la méthode MLST. Les techniques moléculaires ont montré que les souches de sérotype 1 étaient clonales et génétiquement reliées à d’autres souches de sérotype 1 provenant de cas de méningite survenus au Ghana, au Burkina Faso, au Togo et en Egypte. D’autres souches de même sérotype (2, 5 ou 12 par exemple) sont rassemblées dans des complexes clonaux circulant dans différents pays africains. Conclusion. Très peu de sérotypes circulant au Niger sont inclus dans le vaccin conjugué. Les nouvelles techniques de génotypage permettent de suivre l’émergence et la diffusion des nouveaux clones.
M É T H O D E S
 Type d’étude
 Surveillance épidémiologique et microbiologique des méningites bactériennes par le CERMES (Centre National de Référence pour les méningites au Niger) de 2003 à 2006.
 Analyses biologiques
 Détection directe de S. pneumoniae dans le LCR par PCR ± culture ± agglutination latex.
 PCR : amplification du gène lytA (J Clin Microbiol 1996; 34:2030-2).
 Sérotypage.
 Génotypage par MultiLocus Variable Number of Tandem repeats (MLVA), 15 marqueurs, (BMC Microbiology 2005; 5:66) et par Multilocus sequence typing (MLST).
R É S U L T A T S
 Place du pneumocoque dans les méningites bactériennes aiguës au Niger
 Janv. 2003 à déc. 2006 : 16 957 cas suspects de méningite bactérienne aiguë.
 7 204 LCR prélevés (suspicions cliniques de méningite bactérienne aiguë).
 Diagnostic bactériologique obtenu pour 3 155 LCR (44 %) :
 Méningocoque : 	2 517 (80 %)
 Pneumocoque :	 457 (15 %)
 Haemophilus influenzae : 	 142 (5,5 %)
 Variation dans le temps des méningites à pneumocoque au Niger
 Nombres de cas annuels inversement proportionnels à ceux des méningites à méningocoque :
Ex. : 2003 : méningo = 870 cas vs pneumo = 84 cas ; 2005 : méningo = 165 cas vs pneumo = 144 cas
 Nette recrudescence saisonnière de janvier à avril (Figure 1)
 Sexe ratio H:F =1,5:1
 Variation selon l’âge :
 Toutes les classes d’âge sont concernées
 < 2 ans = 30 % des cas
 Taux de létalité : 50 % 
Pneumocoque = cause n° 1 des décès
par méningite bactérienne aiguë au Niger
Nombre
6A
46
2
35F
10F
1
1
1
1
1
1
1
1
1
9
9
9
9
9
9
9
9
9
4
4
4
4
4
4
4
4
4
8
8
8
8
8
8
8
8
8
7
7
7
7
7
7
7
7
7
1
1
1
1
1
1
1
1
1
8
8
8
8
8
8
8
8
8
7
7
7
7
7
7
7
7
7
8
8
8
8
8
8
8
8
8
2
2
2
2
2
2
2
2
2
3
3
3
3
3
3
3
3
3
9
9
9
9
9
9
9
9
9
8
8
8
8
8
8
8
8
8
8
8
8
8
8
8
8
8
8
3
3
3
3
3
3
10
10
10
10
10
10
10
10
10
4
4
4
4
4
7
7
7
7
7
7
7
7
7
7
7
7
7
7
7
7
7
7
6
6
6
6
6
6
6
6
6
1
1
1
1
1
1
1
1
1
3
3
3
3
3
3
3
3
3
8
8
8
8
8
8
8
8
8
24F
2
45
NT
2
35F
B
23F
2
1
1
1
1
1
1
1
1
1
1
1
1
1
1
1
1
1
1
1
1
1
1
1
1
1
1
1
3
3
3
1
1
1
1
1
1
1
1
1
1
1
1
9
9
9
1
1
1
9
9
9
1
1
1
9
9
9
7
7
7
7
7
7
7
7
7
1
1
1
1
1
1
1
1
1
1
1
1
1
1
1
1
1
1
1
1
1
1
1
1
1
1
1
1
1
1
1
1
1
1
1
1
1
1
1
1
1
1
1
1
1
2
2
2
2
2
2
2
2
2
2
2
2
2
2
2
2
2
2
23F
1
1
12F
1
1
23F
14
12B
6
6
6
6
6
6
6
6
6
1
(n=14)
D
14
5
14
4
4
4
4
A
14
12F
3
3
3
3
3
3
3
3
3
1
12F
C
12B
1
12B
18C
1
18C
18C
12B
18C
18C
18C
ST 2 206
ST 63
ST 303
ST 217
ST 74
ST 1 233
ST 289
ST 802
ST 1 368
ST 2 212
ST 989
Figure 1. Cas mensuels déclarés de méningite à pneumocoque, Niger, 2002-2006
 1 cercle = 1 génotype (taille proportionnelle au nb de souches)
 Intérieur du cercle : sérotype
 Distance entre 2 cercles proportionnelle à la proximité génétique
 Fonds colorés = complexes clonaux
 Complexe clonal A : fond rose
Tableau I. Sérotypes des 74 souches de pneumocoque étudiées
| Sérotype | Nb souches |
| --- | --- |
| 1 | 24 |
| 18C | 6 |
| 2, 12B | 5 |
| 14, 23F, 45 | 4 |
| 5, 12F | 3 |
| 48, 35F, 6A | 2 |
| 4, 7C, 10F, 19F, 20, 22A, 24F, 46, | 1 |
Figure 2. Arbre phylogénétique (Minimum Spanning Tree) représentant les différents génotypes MLVA comparés aux génotypes MLST et aux sérotypes des souches de pneumocoque isolées durant la période 2003-2006 au Niger
 Diversité phénotypique et génotypique des souches étudiées
 74 souches réparties au cours de la période d’étude ont été génotypées par MLVA et par MLST.
 Mise en évidence de 51 génotypes MLVA pour les 74 souches. Bonne corrélation avec les sérotypes.
 Très bonne corrélation MLVA – MLST. MLVA plus discriminant.
 Sérotype 1 > 30 % des souches. Toutes les souches de sérotype 1 appartenaient au complexe clonal A, celles de sérotypes 2, 5 ou 12 appartenaient respectivement à d’autres complexes clonaux.
 Le complexe clonal A ne comprenait que des souches de sérotype 1 (à l’exception notable d’une souche de sérotype 23F).
 En 2005 (année où le nombre de cas déclarés était le plus élevé), la diversité génétique était la plus faible : 10 souches sur 16 (63 %) de sérotype 1 et du complexe clonal A.
D I S C U S S I O N - C O N C L U S I O N
Le pneumocoque : une cause majeure de mortalité en Afrique subsaharienne.
Saisonnalité des méningites à pneumocoque dans la ceinture de la méningite.
Pneumocoques de sérotype 1 : cause majeure de méningites purulentes en Afrique.
MLVA : technique discriminante, corrélée au MLST, facile à mettre en oeuvre, peu coûteuse et adaptée à une surveillance microbiologique en temps réel sur le terrain.
Les souches nigériennes de sérotype 1 du complexe clonal A étaient génétiquement reliées aux autres souches de sérotype 1 isolées en Afrique de cas de méningite (Burkina Faso, Togo, Egypte, Kenya). Il semble que toutes ces souches ont une origine commune. D’autres sérotypes sont rassemblés dans des complexes clonaux (B, C et D) circulant dans d’autres pays africains.
Les génotypes des souches de sérotype 1 américaines et européennes sont au contraire génétiquement i) plus diverses et ii) éloignées des souches africaines.
D’autres études montrent également que le caractère épidémique des méningites à pneumocoque est associé à une réduction de la diversité génétique des souches de pneumocoque.
Le vaccin antipneumococcique conjugué actuel (PREVENAR®) contient les sérotypes 4, 6B, 9V, 14, 18C, 19F et 23F et ne couvre donc que 22 % des sérotypes en cause au Niger.
Un vaccin antipneumococcique destiné au continent africain doit absolument inclure le sérotype 1.
La surveillance des sérotypes et des génotypes circulants est nécessaire avant la mise en place de la vaccination et doit se poursuivre après celle-ci pour détecter l’émergence de nouvelles souches.
